# Expression characteristics of peripheral blood miR-146a and TLR4 in septic cardiomyopathy: Correlations with myocardial injury markers and cardiac function classification

**DOI:** 10.12669/pjms.42.5.15866

**Published:** 2026-05

**Authors:** Yejiao Shen, Zheng Zhou, Yan Sha, Lei Nie, Min Gao

**Affiliations:** 1 Yejiao Shen, Department of Emergency, Huashan Hospital, Fudan University, Shanghai 200040, P.R. China; 2 Zheng Zhou, Department of Emergency, Huashan Hospital, Fudan University, Shanghai 200040, P.R. China; 3 Yan Sha, Department of Emergency, Huashan Hospital, Fudan University, Shanghai 200040, P.R. China; 4 Lei Nie Department of Emergency, Tongren Hospital, Shanghai Jiaotong University School of Medicine, Shanghai 200036, P.R. China; 5 Min Gao Department of Nephrology, Tongren Hospital, Shanghai Jiaotong University School of Medicine, Shanghai 200036, P.R. China

**Keywords:** Cardiac function classification, miR-146a, Myocardial injury markers, Septic cardiomyopathy, TLR4

## Abstract

**Objective:**

To investigate the expression levels of peripheral blood microRNA-146a (miR-146a) and Toll-like receptor 4 (TLR4) in patients with septic cardiomyopathy (SCM), and their association with myocardial injury markers and cardiac function classification.

**Methodology:**

This retrospective study included clinical data from 92 patients with SCM who were hospitalized at Shanghai Tongren Hospital between January 2023 and December 2025 (study group). The clinical data of 92 patients with uncomplicated sepsis admitted during the same period were used as the control group. Fasting peripheral venous blood samples were collected from all patients to measure the levels of miR-146a, TLR4, and myocardial injury markers (cTnI, CK-MB, BNP), and to assess cardiac function classification. The levels of miR-146a, TLR4, and myocardial injury markers were compared between the study and control groups. Additionally, miR-146a and TLR4 levels were evaluated in patients with different cardiac function classifications. The correlation between miR-146a, TLR4, myocardial injury markers, and cardiac function classification was further analyzed.

**Results:**

The levels of miR-146a and TLR4 in the study group were higher than those in the control group (miR-146a: 3.26±0.85 vs. 1.05±0.32, P<0.001; TLR4: 68.52±12.36 ng/L vs. 32.18±8.45 ng/L, P<0.001). The levels of cTnI, CK-MB, and BNP in the study group were also higher than those in the control group (cTnI: 0.85±0.26 ng/mL vs. 0.08±0.03 ng/mL, P<0.001; CK-MB: 45.32±10.25 U/L vs. 15.68±4.36 U/L, P<0.001;BNP: 1256.38±325.64 pg/mL vs. 185.42±65.31 pg/mL, P<0.001). There were significant differences in miR-146a and TLR4 levels among patients with different cardiac function classifications (P<0.05). The levels of miR-146a and TLR4 were significantly positively correlated with myocardial injury markers and cardiac function classification. In particular, the correlation coefficients of TLR4 with cTnI and cardiac function classification were 0.702 and 0.817, respectively, while those of miR-146a were 0.718 and 0.823, respectively (all P<0.001).

**Conclusion:**

The expression levels of peripheral blood miR-146a and TLR4 in patients with SCM are significantly elevated, and their expression levels positively correlate with the levels of myocardial injury markers and the severity of cardiac function impairment.

## INTRODUCTION

Sepsis is a systemic inflammatory response syndrome that can, in severe cases, progress to multiple organ dysfunction syndrome.^1^ With mortality rates as high as 30–50%, sepsis remains a critical clinical challenge.^2^,^3^ Septic cardiomyopathy (SCM) is one of the common severe complications of sepsis, characterized primarily by myocardial systolic and diastolic dysfunction, and can significantly increase the disease severity and mortality risk of patients with sepsis.^4^,^5^ At present, the pathogenesis of SCM has not been fully elucidated, and the treatment mainly relies on anti-infective therapy, fluid resuscitation, and symptomatic supportive care, which are often associated with unsatisfactory clinical outcomes.^6^,^7^

MicroRNAs (miRNAs) are a class of non-coding single-stranded RNAs that participate in various physiological and pathological processes, such as the inflammatory response, immune regulation, and cell apoptosis, through targeted regulation of gene expression.^8^ Among them, miR-146a has been shown to be closely associated with the systemic inflammatory response in sepsis and to affect myocardial cell function by regulating downstream target genes.^9^ Toll-like receptor 4 (TLR4) is a key receptor of the innate immune response; its activation can trigger the nuclear factor-κB (NF-κB) signaling pathway, leading to the release of numerous inflammatory factors and subsequent myocardial injury.^10^ Recent studies have shown that miR-146a acts as a crucial negative regulator of TLR4 signaling and regulates TLR-mediated activation of NF-*κ*B through a negative feedback loop in human monocytes.^11^,^12^

This study aimed to investigate the expression characteristics of peripheral blood miR-146a and TLR4 in patients with SCM and to analyze their correlations with myocardial injury markers, such as cardiac troponin I (cTnI), creatine kinase-MB (CK-MB), and brain natriuretic peptide (BNP), as well as with cardiac function classification. The results may provide a new theoretical basis and identify potential biomarkers for the early diagnosis, disease assessment, and targeted therapy of SCM.

## METHODOLOGY

**Fig.1:**
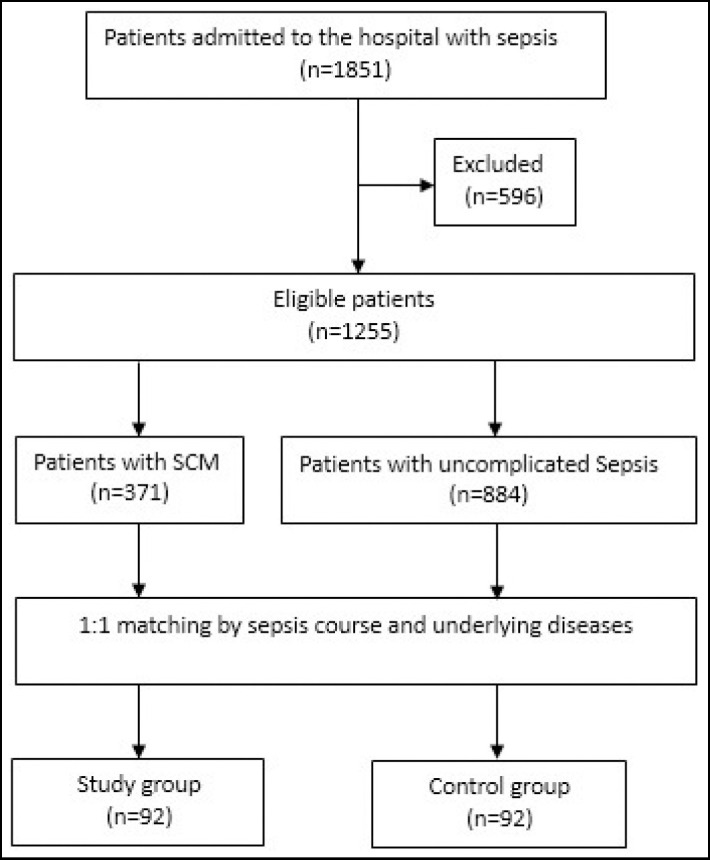
Flowchart of patient selection.

This retrospective study analyzed the clinical data of 184 patients with sepsis admitted to Shanghai Tongren Hospital from January 2023 to December 2025. Of them, 92 patients with SCM were enrolled as the study group, while 92 patients with uncomplicated sepsis (without complicated myocardial injury) admitted during the same period were selected as the control group (Fig.1).

### Ethical approval:

The study was approved by the Ethics Committee of the Shanghai Tongren Hospital (K2025-050-01), Date: July 2, 2025. Due to the retrospective nature of the study, the requirement for informed consent was waived.

### Inclusion Criteria:

- Aged 18 to 80 years, regardless of gender.
- Patients met the diagnostic criteria for sepsis.^13^
- Patients in the study group conforming to the diagnostic criteria for SCM (acute uni- or bi-ventricular systolic or diastolic dysfunction with reduced contractility not due to coronary disease), as confirmed by echocardiography. 14
- Patients in the control group were diagnosed with no myocardial injury based on dynamic observation, combined with echocardiography and detection of myocardial injury markers.
- Time interval from disease onset to hospital admission ≤ 72 hours.
- Complete clinical data available for analysis.

### Exclusion Criteria:

- Patients with acute coronary syndrome, active ischemic cardiomyopathy, primary cardiomyopathy, stress cardiomyopathy, or other pre-existing cardiac conditions that could independently explain the acute cardiac dysfunction were excluded..
- Complicated with malignant tumors, autoimmune diseases, chronic liver or renal failure, or hematological diseases.
- History of major surgery or severe trauma within the past three months.
- Long-term administration of immunosuppressants or high-dose glucocorticoids.
- Pregnant or lactating women.
- Missing key clinical data, which makes it impossible to complete the analysis of relevant indicators.

### Collected Indexes:

Fasting peripheral venous blood samples (5 mL) were collected from all patients within 24 hours of admission. Of this, 2 mL was placed in an ethylenediamine tetraacetic acid (EDTA) anticoagulant tube and centrifuged at 3000 r/min for 15 minutes at 4°C to separate the upper plasma layer, which was then stored at -80°C for later testing. The remaining 3 mL was placed in a tube without an anticoagulant and allowed to coagulate naturally at room temperature for 30 minutes. Afterward, the sample was centrifuged at 3000 r/min for 15 minutes at 4°C to separate the serum, which was then aliquoted and stored at -20°C for the detection of myocardial injury markers and TLR4 protein.

### Detection of miR-146a expression levels by qRT-PCR:

The plasma microRNA extraction kit (B518285, Shanghai Sangon Biotech Co., Ltd.) was used according to the kit instructions. RNA purity (A260/A280=1.8~2.1) and concentration were measured using a NanoDrop 2000 ultramicro spectrophotometer (Thermo Fisher Scientific, USA), and the RNA was stored at -80^0^C. Reverse transcription was carried out using the miRNA first-strand cDNA synthesis kit (B532451, Shanghai Sangon Biotech Co., Ltd.). QPCR amplification was performed using the following oligonucleotides:

- hsa-miR-146a forward: GGAATTCGCTGAGAACTGAATTCCAT,
- hsa-miR-146a reverse: CCGCTCGAGTCCAGTTTTCCCAG, with an amplification product length of 62 bp.
- U6 snRNA forward: CTCGCTTCGGCAGCACA,
- U6 snRNA reverse: AACGCTTCACGAATTTGCGT, with an amplification product length of 96 bp.
- All primers were designed and synthesized by Shanghai Sangon Biotech Co., Ltd.

Quantitative polymerase chain reaction (qPCR) was done using the SYBR® Green qPCR Mix reagent kit (B532461, Shanghai Sangon Biotech Co., Ltd.). The amplification was performed using a LightCycler 480 II real-time qPCR instrument (Roche, Switzerland). Each sample was run in triplicate, and a blank control (without cDNA template) was also included. The specific amplification conditions were as follows: a pre-denaturation phase at 95°C for 30 s; a PCR cycle phase consisting of 40 cycles, each cycle including a denaturation step at 95°C for 5 s and an annealing and extension step at 60°C for 30 s, with fluorescence signals collected simultaneously during the annealing and extension phase; a melting curve analysis phase consisting of 95°C for 10 s and 65°C for one minute, followed by continuous fluorescence collection at 97°C to confirm the specificity of the amplification product. Finally, the relative expression level of miR-146a was calculated using the 2^-ΔΔCt method, with U6 snRNA as the internal reference gene. The experimental data were averaged across the three replicates.

### Detection of TLR4 protein expression level:

It was done by the enzyme-linked immunosorbent assay (ELISA) using the TLR4 ELISA kit (SEKH-0014, Beijing Senbeijia Biotechnology Co., Ltd.). A Multiskan FC automatic microplate reader by Thermo Fisher Scientific (USA) was used to measure absorbance (OD) at 450 nm.

### Detection indicators and methods for myocardial injury markers:

The levels of cardiac troponin I (cTnI) and creatine kinase-MB (CK-MB) in serum were measured using an automatic biochemical analyzer. The level of B-type natriuretic peptide (BNP) was detected using a chemiluminescence immunoassay method. All operations were conducted strictly in accordance with the instructions provided by the instrument and the corresponding reagent kits.

The cardiac function of patients was graded according to the New York Heart Association (NYHA) classification criteria. Patients were categorized into four classes: *Class- I:* No limitation of physical activity; *Class-II:* Slight limitation of physical activity; *Class-III:* Marked limitation of physical activity; *Class-IV:* Inability to carry out any physical activity without discomfort, and heart failure symptoms are present even at rest. Cardiac function classification was determined based on clinical records, including symptoms, hemodynamic status, and physician assessment during hospitalization. For patients unable to report symptoms (e.g., due to sedation or mechanical ventilation), classification was based on objective clinical indicators and documented functional status prior to clinical deterioration.

Levels of miR-146a and TLR4 were compared between the study and control groups, and among study-group patients with different cardiac function classifications. Levels of myocardial injury markers (cTnI, CK-MB, BNP) were compared between the study and control groups. The correlations between the levels of miR-146a and TLR4, and the levels of myocardial injury markers (cTnI, CK-MB, BNP) as well as cardiac function classification in the study group were assessed.

### Statistical analysis:

All data analyses were performed using SPSS 26.0. Measurement data were expressed as mean ± standard deviation (). An independent-samples t-test was used to compare two groups, while a one-way analysis of variance (ANOVA) followed by a least significant difference (LSD) post hoc test was used to compare multiple groups. Categorical data were expressed as rates (%). The Chi-square (*χ²*) test was used to compare categorical data between groups. Spearman’s rank correlation coefficient was used to analyze the correlations between the levels of miR-146a and TLR4, and the levels of myocardial injury markers (cTnI, CK-MB, BNP) as well as cardiac function classification. P<0.05 was considered statistically significant.

## RESULTS

This retrospective study included 184 patients. Of them, 92 patients were in the study group (51 males and 41 females, aged 35–78 years, with a mean age of (56.72±8.45) years), and 92 patients were in the control group (49 males and 43 females, aged 34–79 years, with a mean age of (55.96±8.71) years). As shown in Table-I, there were no statistically significant differences in the baseline data between the two groups (P>0.05), indicating good comparability. miR-146a and TLR4 levels in the study group were significantly higher than in the control group (P<0.05) (Table-II). Similarly, the levels of cTnI, CK-MB, and BNP in the study group were significantly higher than those in the control group (P<0.05) (Table-III).

**Table-I:** Comparison of baseline data between the two groups.

Group	No. of Cases	Age (years)	Gender (Male/Female)	Body Mass Index(kg/m²)	Sepsis Course(d)	Underlying diseases			
						Hypertension	Diabetes	Chronic Heart Failure	Chronic Kidney Disease
Study Group	92	56.72± 8.45	51/41	24.18± 2.05	3.26± 1.12	38	29	15	8
Control Group	92	55.96± 8.71	49/43	24.32± 1.98	3.35± 1.20	36	31	13	7
*t/χ² value*		0.601	0.088	0.471	0.526	0.090	0.099	0.169	0.073
*P value*		0.549	0.767	0.638	0.600	0.764	0.753	0.681	0.788

**Table-II:** Comparison of miR-146a and TLR4 levels between the study group and the control group (*x̄*+*s*).

Group	Cases	miR-146a	TLR4(ng/L)
Study group	92	3.26±0.85	68.52±12.36
Control group	92	1.05±0.32	32.18±8.45
*t value*		23.339	23.280
*P value*		<0.001	<0.001

**Table-III:** Comparison of myocardial injury marker levels between the study group and the control group(*x̄*+*s*).

Group	Cases	cTnI(ng/mL)	CK-MB(U/L)	BNP(pg/mL)
Study group	92	0.85±0.26	45.32±10.25	1256.38±325.64
Control group	92	0.08±0.03	15.68±4.36	185.42±65.31
*t value*		28.219	25.523	30.929
*P value*		<0.001	<0.001	<0.001

Among the 92 patients in the study group, 31 were classified as NYHA class II, 38 as class III, and 23 cases as class IV (Table-IV). There were significant differences in miR-146a and TLR4 levels among patients with different cardiac function classifications (P<0.05) (Table-IV). Furthermore, the levels of miR-146a and TLR4 in patients with class III cardiac function were significantly higher than those in patients with class II, while the levels in patients with class IV were significantly higher than those in patients with class III (P<0.05).

**Table-IV:** Comparison of miR-146a and TLR4 levels in study group patients with different cardiac function classifications (*x̄*+*s*).

Group	Cases	miR-146a	TLR4(ng/L)
NYHA Class-II	31	2.15±0.52	52.36±9.85
NYHA Class-III	38	3.58±0.76	72.45±11.23
NYHA Class-IV	23	4.82±0.91	95.68±13.52
*F value*		89.594	95.299
*P value*		<0.001	<0.001

Spearman’s rank correlation analysis confirmed that miR-146a and TLR4 levels were significantly positively correlated with myocardial injury markers (cTnI, CK-MB, BNP) and cardiac function classification (P<0.05) (Table-V).

**Table-V:** Correlations of miR-146a and TLR4 with myocardial injury markers and cardiac function classification.

Index		cTnI	CK-MB	BNP	Cardiac Function Classification
miR-146a	ρ value	0.718	0.704	0.685	0.823
	P value	<0.001	<0.001	<0.001	<0.001
TLR4	ρ value	0.702	0.691	0.676	0.817
	P value	<0.001	<0.001	<0.001	<0.001

## DISCUSSION

This retrospective study showed abnormally high expression of miR-146a and TLR4 in patients with SCM, as well as a significant positive correlation between miR-146a and TLR4 levels and myocardial injury markers and cardiac function classification. The results provide new clinical evidence for the assessment research of SCM.

SCM is a severe complication of sepsis-induced multiple organ dysfunction, and was shown to increase the mortality rate by 2–3 times compared with uncomplicated sepsis.^15^ However, as there are currently no early specific biomarkers of SCM, diagnosis primarily relies on myocardial injury markers and echocardiography, which are susceptible to interference from factors such as underlying heart disease and infection severity.^16^ Therefore, exploring specific and sensitive biomarkers has become a core direction in the clinical research of SCM.

As a key microRNA regulating inflammatory responses, miR-146a can negatively regulate the NF-κB signaling pathway by targeting molecules such as tumor necrosis factor receptor-associated factor 6 (TRAF6) and interleukin-1 receptor-associated kinase 1 (IRAK1), thereby contributing to the balance of innate immunity and inflammatory responses.^17^ The results of this study showed that the expression level of miR-146a in the peripheral blood of patients with SCM was significantly higher than that in patients with uncomplicated sepsis (P<0.05). This result is consistent with the previous report by An R et al,^18^ which showed that in heart-derived H9C2 myocardial cells with induced sepsis, miR-146a expression increased with the progression of myocardial injury. Furthermore, exogenous supplementation of miR-146a reduced cardiomyocyte apoptosis by inhibiting inflammatory responses.^18^ This study further confirmed a positive correlation between peripheral blood miR-146a expression and cardiac function classification (P<0.05), suggesting that peripheral blood miR-146a can serve as a non-invasive indicator of myocardial injury.

TLR4 is an important pattern recognition receptor in the innate immune response; its activation can trigger the NF-κB signaling pathway and release large amounts of pro-inflammatory cytokines, including tumor necrosis factor-α (TNF-α) and interleukin-6 (IL-6), leading to cardiomyocyte edema, apoptosis, and systolic dysfunction.^19^ In this study, TLR4 levels in patients with SCM were significantly elevated (P<0.05), consistent with the clinical research findings of Liu Q et al.^20^ The study found that peripheral blood TLR4 levels in patients with myocardial injury were significantly higher than in those without, and that TLR4 levels were positively correlated with in-hospital mortality. In conjunction with previous reports, this study confirmed that excessive activation of the TLR4-mediated inflammatory signaling pathway is an important mechanism underlying sepsis-induced myocardial injury.

The levels of myocardial injury markers (cTnI, CK-MB, BNP) in the study group were significantly higher than those in the control group (P<0.05), confirming that patients with SCM have definite myocardial injury and cardiac dysfunction. Elevated levels of TnI, a cardiomyocyte-specific marker, indicate damage to the integrity of cardiomyocytes, while CK-MB can reflect the severity of myocardial injury, and BNP is closely related to ventricular wall tension and serves as a core indicator for evaluating cardiac function status.^21^

This study found significant differences in miR-146a and TLR4 levels among patients with different cardiac function classifications within the study group, with levels gradually increasing as the NYHA classification increased (P<0.05). Mechanistically, a higher cardiac function classification indicates more severe myocardial inflammatory injury: the body upregulates miR-146a expression to enhance anti-inflammatory compensatory effects, while the continuous elevation of TLR4 reflects the persistent activation of inflammatory signaling pathways.^22^,^23^ The dynamic changes of these two molecules together constitute a molecular marker for the progression of myocardial injury.

Recent research has shown that miRNA-146a improves sepsis-induced cardiomyopathy by regulating the TLR-4/NF-κB signaling pathway.^24^ However, while previous studies have primarily investigated miR-146a or TLR4 individually in the context of sepsis-induced myocardial injury, there is limited evidence evaluating their combined expression in patients with septic cardiomyopathy. The present study showed that miR-146a and TLR4 levels were elevated in patients with SCM and increased progressively across higher cardiac function classes, supporting their association with the severity of cardiac dysfunction. These findings are consistent with previous evidence that miR-146a and TLR4-related signaling are involved in innate immune responses and sepsis-induced myocardial dysfunction.^25^,^26^ Therefore, miR-146a and TLR4 may serve as potential biomarkers for assessing myocardial injury and disease severity in patients with SCM. However, their diagnostic accuracy and specificity require further validation using formal diagnostic performance analyses. From a clinical perspective, correlations revealed by this study suggest that the combined biomarker approach may be most applicable for severity stratification rather than early screening, while also providing a basis for future mechanistic studies exploring the miR-146a/TLR4 regulatory axis in SCM.

### Limitations:

First, as a single-center retrospective study with a small sample size, the selection bias may affect the extrapolation of the results. Second, the occurrence and severity of SCM are influenced by many important clinical factors, but only several baseline factors are compared across groups, which may result in the study’s findings being partially confounded by unmeasured factors. Third, this study only explored the expression characteristics and correlations between miR-146a and TLR4, without in-depth analysis of their specific mechanisms of action and upstream and downstream regulatory relationships. In addition, the study did not conduct long-term prognostic follow-up of the patients, so it is impossible to clarify the association between the expression levels of these two molecules and patient mortality or cardiac function recovery. The lack of long-term follow-up data may limit the generalizability of the findings to patients at different disease stages or severity levels, rendering them inappropriate as a basis for risk prediction or intervention decision-making in clinical practice. Additionally, this study used the NYHA classification to stratify cardiac function. Although NYHA classification is widely applied in both chronic and acute heart failure settings,^27^,^28^ it is primarily a symptom-based functional assessment and may have limited applicability in ICU patients with SCM, who may require sedation, mechanical ventilation, or strict bed rest. In such cases, functional status cannot be reliably assessed based solely on patient-reported symptoms, and classification may rely on indirect clinical judgment or surrogate indicators. Therefore, the observed associations between miR-146a, TLR4, and NYHA class should be interpreted as reflecting the relative severity of cardiac dysfunction rather than precise functional capacity.

## CONCLUSION

This study demonstrated that the expression levels of peripheral blood miR-146a and TLR4 in patients with SCM are significantly elevated and positively correlate with the levels of myocardial injury markers and the severity of cardiac function impairment. These findings suggest that miR-146a and TLR4 may be involved in the pathogenesis of SCM and can serve as potential biomarkers for assessing the extent of myocardial injury and cardiac function in patients.

### Recommendations:

Further multicenter, large-sample prospective studies are needed to further verify the clinical value of miR-146a and TLR4. *In vitro* and animal model studies should explore the regulatory mechanisms underlying SCM. More objective measures of cardiac function, such as echocardiographic parameters or hemodynamic indices, are needed. The predictive value of miR-146a and TLR4 should be combined with prognostic follow-up data to provide a more comprehensive theoretical basis for early diagnosis, disease assessment, and targeted therapy of SCM.

### Author’s contributions:

**YS and ZZ:** Literature search, study design and manuscript writing.

**Yan Sha, LN and MG:** Data collection, data analysis and interpretation. Critical review.

**YS and ZZ:** Manuscript revision and validation and is responsible for the integrity of the study.

All authors have read and approved the final manuscript.
